# Structural insight into the assembly and conformational activation of human origin recognition complex

**DOI:** 10.1038/s41421-020-00232-3

**Published:** 2020-11-24

**Authors:** Jiaxuan Cheng, Ningning Li, Xiaohan Wang, Jiazhi Hu, Yuanliang Zhai, Ning Gao

**Affiliations:** 1 State Key Laboratory of Membrane Biology, School of Life Sciences, Tsinghua University, Beijing 100084, China; 2 State Key Laboratory of Membrane Biology, Peking-Tsinghua Joint Center for Life Sciences, School of Life Sciences, Peking University, Beijing 100871, China; 3 School of Biological Sciences, The University of Hong Kong, Hong Kong, China

**Keywords:** Cryoelectron microscopy, Origin selection

## Abstract

The function of the origin recognition complex (ORC) in DNA replication is highly conserved in recognizing and marking the initiation sites. The detailed molecular mechanisms by which human ORC is reconfigured into a state competent for origin association remain largely unknown. Here, we present structural characterizations of human ORC1–5 and ORC2–5 assemblies. ORC2–5 exhibits a tightly autoinhibited conformation with the winged-helix domain of ORC2 completely blocking the central DNA-binding channel. The binding of ORC1 partially relieves the autoinhibitory effect of ORC2–5 through remodeling ORC2-WHD, which makes ORC2-WHD away from the central channel creating a still autoinhibited but more dynamic structure. In particular, the AAA+ domain of ORC1 is highly flexible to sample a variety of conformations from inactive to potentially active states. These results provide insights into the detailed mechanisms regulating the autoinhibition of human ORC and its subsequent activation for DNA binding.

## Introduction

In eukaryotes, DNA replication initiation is tightly regulated to ensure that the genome can be replicated once and only once per cell cycle. Central to this regulation is the loading and subsequent activation of the mini-chromosome maintenance (Mcm2–7) helicase complex at replication origins^1–3^. The origin recognition complex (ORC), composed of Orc1–6, is a highly conserved heterohexameric complex, which plays a critical role in recognizing origin DNA and promoting helicase loading. Each of the Orc1–5 subunits bears an AAA+ or AAA+-like domain and an α-helical winged-helix domain (WHD). The AAA+ domains can be further decomposed into a RecA subdomain and an α-helical lid subdomain (Supplementary Fig. S1a). Among Orc1–5, Orc1, Orc4, and Orc5 each contains a functional AAA+ domain for nucleotide binding; in contrast, Orc2 and Orc3 only contain a RecA-like subdomain with no predicted ATPase activities^4–6^. Orc6 bears little resemblance to other Orc subunits but is essential for helicase loading^5,7–9^.

According to studies in yeast, ORC recognizes and binds to replication origins in an adenosine triphosphate (ATP)-dependent manner in G1 phase^4,10^. After Cdc6 (Cell division cycle 6) incorporation, ORC–Cdc6 serves as a platform to recruit two copies of the hetero-hexameric Mcm2–7 helicase complex, one at a time and with the help of Cdt1 (Cdc10-dependent transcript 1), to form a head-to-head double hexamer (DH) encircling dsDNA^11,12^. This process is also known as pre-replication complex (pre-RC) assembly. The DH remains inactive in its helicase activity throughout the remaining G1 phase. Upon S phase entry, two kinases, DDK (Dbf4-dependent kinase) and S-CDK (S-phase-specific cyclin-dependent kinase), act in concert with multiple initiation factors to transform the DH into two active Cdc45–Mcm2–7–GINS (CMG) helicases^13–15^. This process also accompanies with origin DNA melting as well as reconfiguration of the CMG from encircling dsDNA to capturing only ssDNA for helicase translocation along leading-strand DNA. As a result, two active replisomes can be formed at replication forks for bidirectional DNA synthesis^16,17^.

High-resolution structures of *Drosophila melanogaster* ORC (DmORC)^18^, *Saccharomyces cerevisiae* ORC (ScORC)–DNA complex^19^, ScORC–Cdc6–Cdt1–MCM (OCCM) complex^20^, and two human ORC (HsORC) subcomplexes, ORC1/4/5 and ORC2/3^21^ have been recently reported. These structures together revealed a highly conserved architecture for eukaryotic ORC complexes, and suggested that continuous structural rearrangements in different ORC subunits are required to shape the complex into an active and open state for DNA binding and Cdc6 incorporation^18,19,21^.

Despite the above conserved features, the activities of ORC in DNA binding are markedly different among various species. It is known that ScORC has a strict requirement for a specific ARS consensus sequence (ACS)^4,19,22^. In contrast, DNA binding of metazoan ORC is predominantly determined by specifically modified nucleosomes and particular chromatin structures rather than DNA sequence alone^23–27^, and also involves a number of diverse accessory proteins^28–34^. Once bound to replication origin, ScOrc1–6 stays as an intact assembly at origin DNA throughout the cell cycle^35–37^. In contrast, HsORC only associates with replication origins in G1 phase, and the assembly of HsORC at origin DNA is regulated in a stepwise manner, with ORC1 being the first to be recruited onto chromatin^27,38,39^. In human cells, ORC2–5 forms a stable core subcomplex with ORC1 and ORC6 loosely attached^40–42^. However, the targeting of ORC2–5 onto chromatin is ORC1-dependent^38,40,41,43^. It has been shown that the nuclear import of ORC1 and ORC6 is also separate from ORC2–5^40,41^. When cell enters S phase, ORC1 dissociates from ORC2–5 and is released from chromatin before being degraded by a ubiquitin-proteasome pathway^43–46^. In parallel, ORC2 phosphorylation strips off ORC2–5 from chromatin^47^. Different from ORC1, the cellular level of ORC2–5 subcomplex remains relatively constant throughout the cell cycle^38,39^. Away from DNA, metazoan ORC resides in an autoinhibited conformation with a closed and constricted central channel to preclude DNA binding^18,48^. These regulations may serve as a safeguard mechanism to prevent pre-RC re-assembly outside the G1 phase.

It is believed that the assembly of metazoan ORC at origin DNA involves a series of conformational re-arrangements in various ORC subunits, leading to an activated initiator complex ready for DNA binding and helicase loading. So far, the detailed mechanisms for ORC activation are not well understood at molecular level. In this study, we determined the cryo-EM structures of HsORC2–5 and HsORC1–5. Structural comparisons indicate that HsORC2–5 also adopts an autoinhibited conformation but with the central DNA-binding channel completely blocked by ORC2-WHD. The binding of ORC1 reconfigures ORC2-WHD in a way creating a slightly expanded but still sealed channel in ORC1–5 that occludes DNA entry. In HsORC1–5, a highly flexible ORC1-AAA+ is able to transit between inhibited and active states. These data help to provide insights into the activation of metazoan ORC for its recruitment onto origin DNA through a step-wise conformational reconfiguration.

## Results

### Structural determination of the HsORC2–5 and HsORC1–5 complexes

To prepare recombinant HsORC samples, all six ORC subunits (ORC1 to ORC6) in full-length were co-overexpressed in insect expression system. The HsORC in soluble cell lysate was then affinity-purified with anti-Flag immunoprecipitation of N-terminal Flag-tagged ORC2 (Supplementary Fig. S1b, d). ORC1 and ORC6 co-purified with ORC2 were in much lower amount when compared with other ORC subunits.

It was previously reported that almost all stable ORC1–6 complexes are associated with chromatin in human cells^38–41,45^. In order to acquire sufficient ORC samples with ORC1 incorporated, an additional high-salt incubation was applied to cell lysates to release ORC from chromatin (Supplementary Fig. S1c; Materials and methods). After this treatment, the amount of purified ORC subunits, especially ORC1, was substantially improved (Supplementary Fig. S1e). A strategy of two-step affinity purification was adopted to further improve the homogeneity of ORC1–5 complex (Supplementary Fig. S1e, g).

The purified ORC2–5 and ORC1–5 samples were further subjected to mild fixation by GraFix^49^ to facilitate cryo-grid preparation (Supplementary Figs. S1b–g, S2, S3). The cryo-EM structures of ORC2–5 and ORC1–5 were determined at resolutions of 3.8 and 4.4 Å, respectively (Supplementary Figs. S2 and S3). One observation during image processing of these datasets is that HsORC appears to be highly dynamic: floppy parts were observed on images of many 2D class averages (Supplementary Fig. S2c), indicating the presence of different forms of subcomplexes. In addition, 3D classification of ORC2–5 particles always resulted in classes with comparable particle numbers (Supplementary Fig. S2d), indicating an intrinsic conformational flexibility. Especially, we noticed that the compositional and conformational heterogeneity for the complex of ORC1–5 is even severer (Supplementary Fig. S3a–c).

These observations are in consistent with the fact that HsORC is highly dynamic^1,21,43^, and suggest that ORC1 further increases both the compositional and conformational heterogeneity. A potential contributing factor is the predicted intrinsic disordered region (IDR) located between the N-terminal Bromo-Adjacent Homology domain (BAH) and AAA+ domain of ORC1, which was recently reported to be able to induce phase separation of the complex^50^. Therefore, the full-length ORC1 is likely the main factor limiting the resolution of our structures (Supplementary Fig. S3d).

### Human ORC2–5 adopts a tightly autoinhibited conformation

**Fig. 1:**
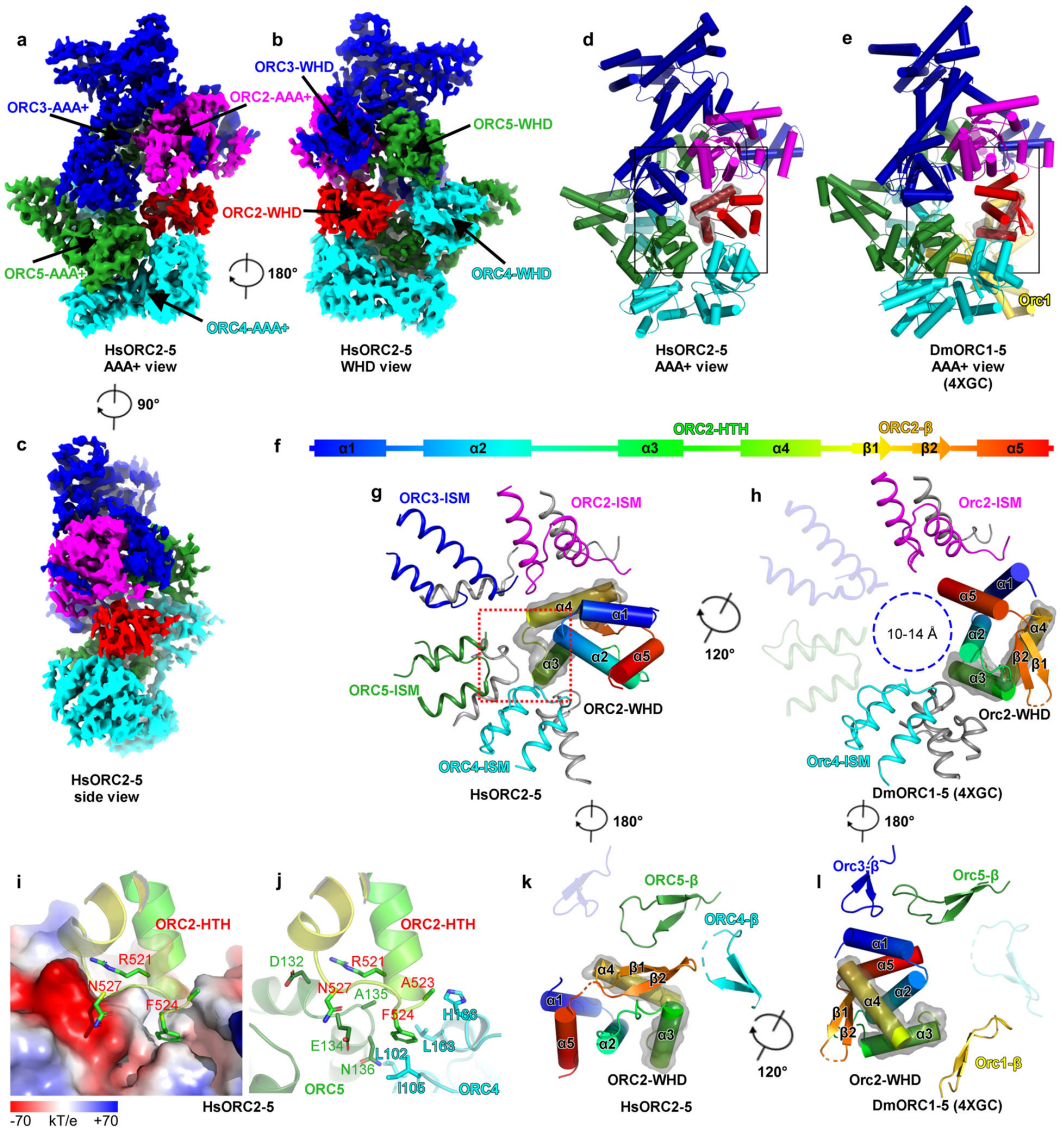
Cryo-EM structure of human ORC2–5. **a**–**c** Cryo-EM density map of HsORC2–5 displayed in AAA+ view (**a**), WHD view (**b**), and side view (**c**). ORC subunits are color-coded and labeled. **d**, **e** Atomic models of HsORC2–5 (**d**) and DmORC (PDB: 4XGC) (**e**) displayed in AAA+ view. Domains are colored in the same color code as in **a**–**c**. **f** Schematic representation of the secondary structural elements of HsORC2-WHD. Secondary structural elements are labeled and colored in rainbow-mode. **g**, **h** Zoomed-in view of the boxed regions in **d**, **e**, showing the interaction between HsORC2-WHD and the AAA+ layer of the other subunits in HsORC2–5 (**g**) and DmORC (**h**). Secondary structure elements of HsORC2-WHD are labeled and colored, and HTH motif (α3–α4) are highlighted with transparent surface representation. For simplification, only the ISM motifs and other motifs (colored gray) interacting with HsORC2-WHD are displayed. ISM motifs are colored in the same color code as in **d**, **e**. ISM motifs without interaction with HsORC2-WHD are set transparent. Blue dashed circle shows the hollow DNA-binding central channel with a diameter of 10–14 Å in **h**. **i**, **j** Zoomed-in view of the boxed region in **g** showing the interaction between the turn loop of HsORC2-HTH motif and the AAA+ domains of HsORC4 and HsORC5. The electrostatic potential surface of HsORC4 and HsORC5 contacting HsORC2-HTH is displayed in **i**. Key residues involved in the interactions are labeled. **k**, **l** Similar to **g**, **h**, but with the view angle rotated 180°, the interaction between HsORC2-WHD and the WHD layer of the other subunits is shown in HsORC2–5 (**k**) and DmORC (**l**). For simplification, only the β-hairpin motifs are displayed and colored in the same color code as in **d**, **e**. β-hairpin motifs without interaction with HsORC2-WHD are set transparent. There is a rotation, ~120°, between the position of HsORC2-WHD in HsORC2–5 and DmOrc2-WHD in DmORC.

The map of ORC2–5 at 3.8-Å resolution allows us to build an atomic model for most parts of ORC2–5 subunits (Supplementary Figs. S1a, S2d, e, and S4 and Table S1), including ORC5-WHD that was trimmed in the previous HsORC1/4/5 crystal structure^21^. Without ORC1, subunits of ORC2–5 still engage with each other in a domain-swapping configuration and form a two-layered structure (AAA+ and WHD layers), similar to yeast and *Drosophila* ORC structures (Fig. 1a–c and Supplementary Fig. S5a–d)^18–20^.

With these general similarities, however, ORC2-WHD displays a very different conformation from all previously reported structures. Compared with DmORC, ORC2-WHD in HsORC2–5 rotates ~120° and resides right in the middle of the ORC ring, completely occupying the central DNA-binding channel (Fig. 1d–h, k, l). This specific conformation is established through an extensive interface between ORC2-WHD and the inner surface of the ORC ring (Supplementary Fig. S5e–j), involving all secondary structural elements of ORC2-WHD. The most significant contribution to this interface appears to be from the helix-turn-helix (HTH) motif (α3 and α4) of ORC2-WHD (Fig. 1d, g, k and Supplementary Fig. S5j). The loop region of this HTH is positioned very close to the cleft between AAA+ domains of ORC4 and ORC5 (Fig. 1i, j) with its Phe524 docked into a hydrophobic pocket formed by the residues from ORC4-RecA (Ile105, Leu163, His166) and ORC5-RecA (Ala135, Asn136) (Fig. 1i, j). In addition, Arg521 and Asn527 of ORC2 form hydrogen bonds with Asp132 and Glu134 of ORC5, respectively (Fig. 1i, j). Moreover, most of the DNA binding motifs of ORC subunits, such as the initiation-specific motif (ISM) from RecA subdomain and the β-hairpin motif from WHD, are blocked by these interactions (Fig. 1g, k and Supplementary Fig. S5b, d). Together, this special arrangement of HsORC2–5 exhibits a tightly inhibited state, excluding DNA entry into the ORC ring. In contrast, although DmORC structure also assumes an autoinhibited conformation, its central channel is not fully occupied by ORC2-WHD, leaving a hollow ring with a diameter of 10–14 Å (Fig. 1h, l).

### ORC1-WHD triggers the first step of ORC conformational activation

**Fig. 2:**
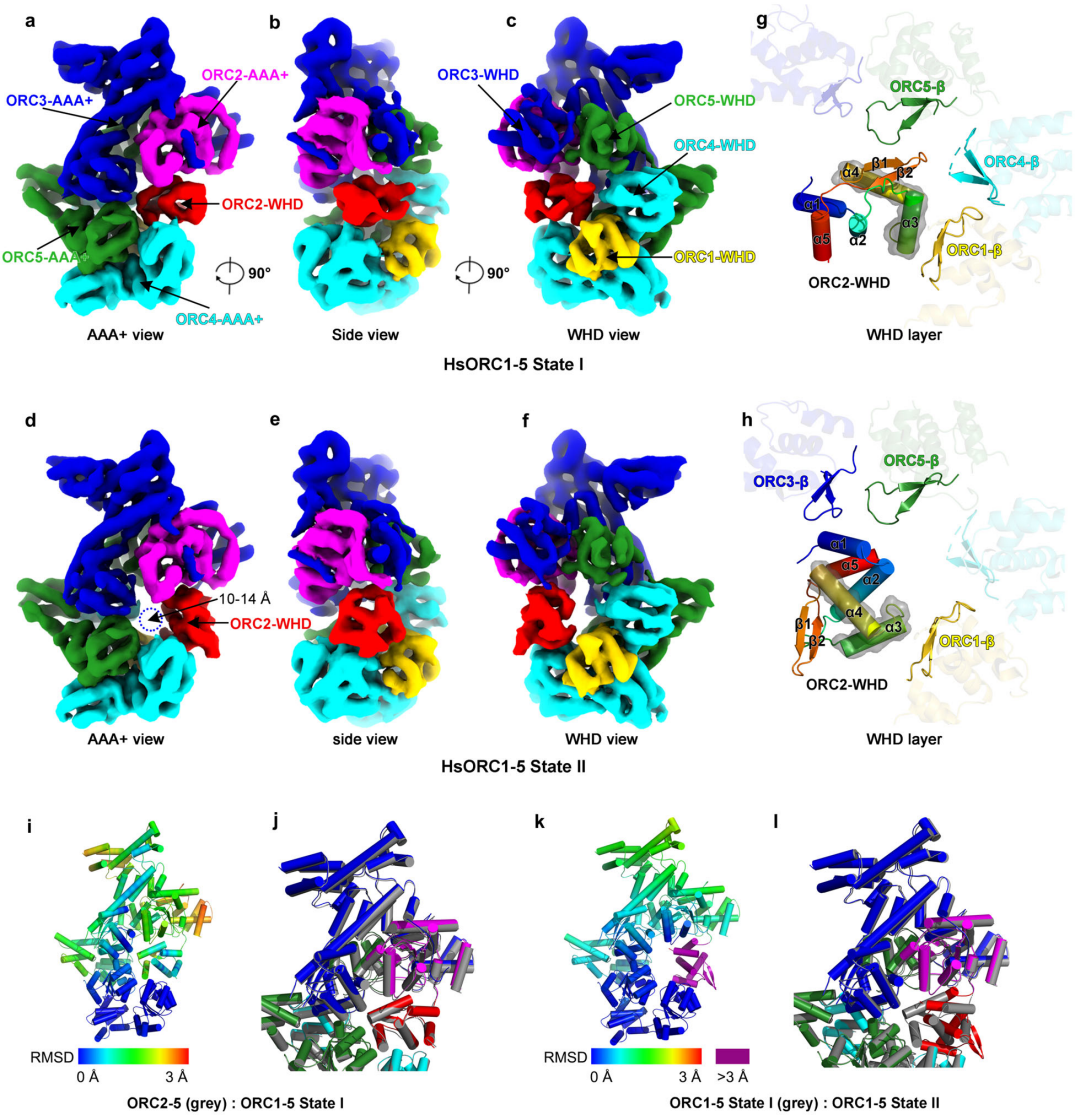
Structural comparison between conformational State I and II of human ORC1–5. **a**–**f** Cryo-EM density maps of HsORC1–5 in State I (**a**–**c**) and II (**d**–**f**), displayed in AAA+ view (**a**, **d**), side view (**b**, **e**) and WHD view (**c**, **f**). ORC subunits are color-coded and labeled. Blue dashed circle shows the hollow DNA-binding central channel with a diameter of 10–14 Å in **d**. **g**, **h** Interactions between ORC2-WHD and WHD layer of the other subunits in States I (**g**) and II (**h**) displayed in the same view as in **c**, **f**. Secondary structure elements of ORC2-WHD are labeled and colored in rainbow-mode, and the HTH motifs are highlighted with transparent surface representation. For simplification, β-hairpin motifs without interactions with ORC2-WHD are set transparent. **i** Temperature map showing the conformational change between ORC2–5 and ORC1–5 State I. ORC2–5 is colored by RMSD between the two states. RMSD is calculated based on the C-alpha atom pairs after alignment using ORC4-RecA as the reference. Color bar is labeled. **j** Superimposition of ORC2–5 and ORC1–5 State I showing the detailed conformational difference. The two structures are aligned based on ORC4-RecA. **k** Same as **i**, conformational change is shown between ORC1–5 State I and II. Color bar is labeled and residues with RMSD more than 3 Å are colored magenta. **l** Same as **j**, superimposition of ORC1–5 State I and II structures are shown.

The structure of HsORC1–5 was solved at a resolution of 4.4 Å (Supplementary Fig. S3). The overall architecture of HsORC1–5 is almost the same as HsORC2–5. While ORC1-WHD is located in a similar position as seen in previous ORC structures (Supplementary Fig. S6a–d), ORC1-AAA+ is not as stable as that in the crystal structure of DmORC (Supplementary Figs. S3b and S6e, f). In addition, the occupancy and the local resolution of ORC2-WHD in the HsORC1–5 map are significantly lower than those in the map of HsORC2–5, suggesting that ORC2-WHD becomes dynamic or has multiple conformations in HsORC1–5 (Supplementary Figs. S3b, c and S6g, h, k, l). This is also evident in the 2D class averages (Supplementary Fig. S7a, b). Careful analysis of the density map suggested that ORC2-WHD in HsORC1–5 is probably a mixture of two conformational states, resembling those in the tightly autoinhibited HsORC2–5 and in the loosely autoinhibited DmORC. With the structures of HsORC2–5 and DmORC as references, a supervised local 3D classification enabled a clear separation of two conformational states, I and II (Fig. 2a–f and Supplementary Figs. S3b and S7c, d). In State I (~40% particles, 5.0 Å resolution), ORC2-WHD is located at a position similar as seen in HsORC2–5 (Fig. 2a–c and Supplementary Figs. S3b and S7a, c and Table S1). In contrast, in State II (~60% particles, 4.8 Å), ORC2-WHD is relocated to an equivalent position of DmORC (Fig. 2d–f and Supplementary Figs. S3b and S7d and Table S1). Interestingly, as observed in the 2D averages, although it is still flexible in both states, ORC1-AAA+ becomes slightly ordered in State II (Supplementary Fig. S7b–d).

**Fig. 3:**
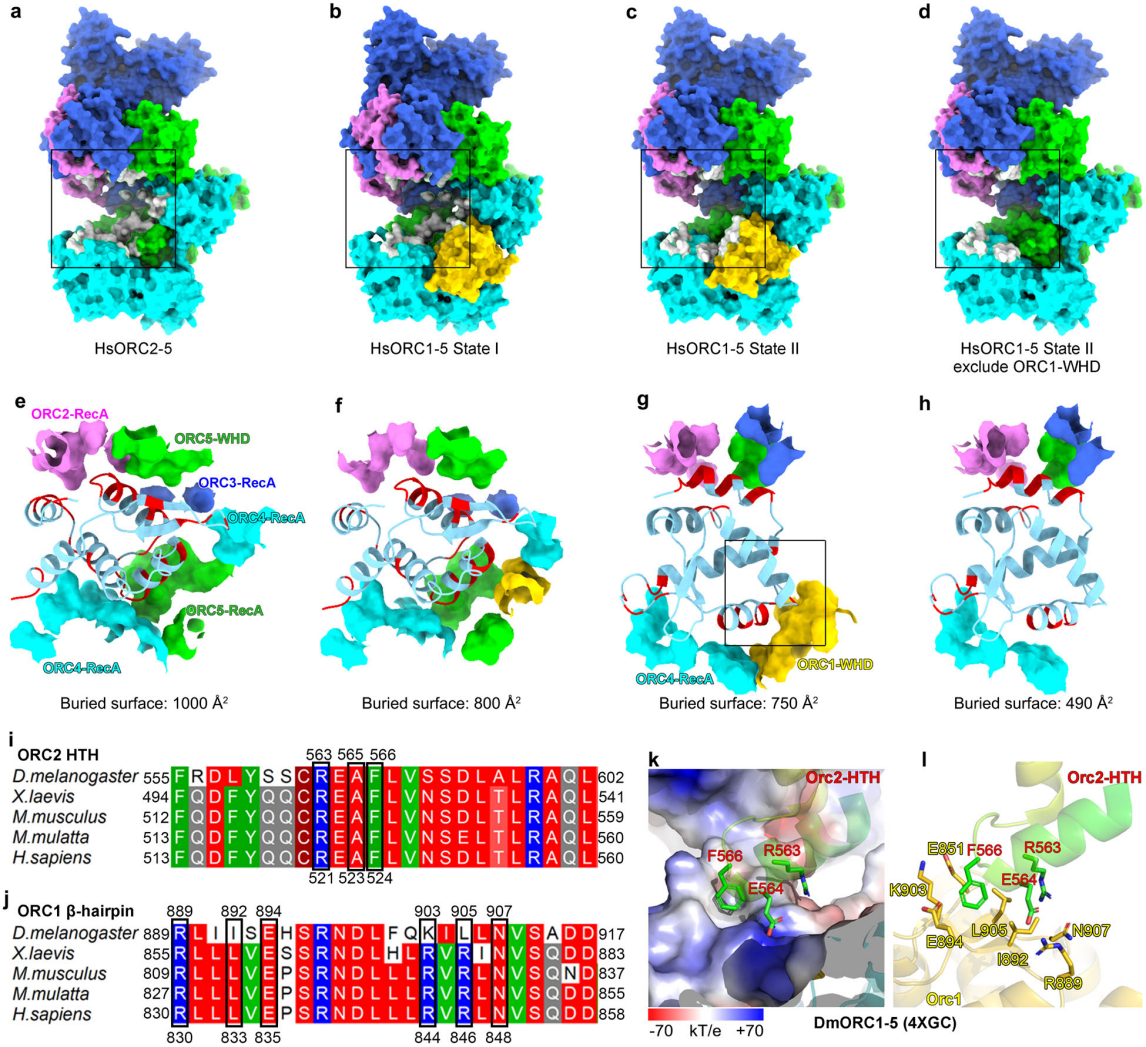
Alteration in the interface between ORC2-WHD and the DNA-binding channel in State I–II transition of human ORC1–5. **a**–**d** Surface representation of the atomic models showing the buried surface of the interface between ORC2-WHD and the DNA-binding channel in ORC2–5 (**a**), ORC1–5 State I (**b**), ORC1–5 State II (**c**) and ORC1–5 State II excluding ORC1–WHD (**d**). ORC subunits are color-coded and labeled, with the buried residues from the channel components highlighted in gray. For clarity, ORC2-WHDs are omitted. **e**–**h** Zoomed-in view of the boxed regions in **a**–**d** showing the detailed interfaces. The buried residues of each domain constituting the DNA-binding channel are extracted and shown as surface. ORC2-WHD is shown in cartoon representation, with corresponding buried residues highlighted in red. **i** Sequence alignment of ORC2-HTH from different species. The key residues contributing to the interaction with β-hairpin of ORC1-WHD are highlighted and numbered. **j** Sequence alignment of ORC1-β-hairpin from different species. The key residues contributing to the interaction with ORC2-HTH are highlighted and numbered. **k**, **l** Atomic interactions between the turn loop of Orc2-HTH and the Orc1-β-hairpin of DmORC (PDB: 4XGC) in the same region boxed in **g**. The electrostatic potential surface of DmOrc1-β-hairpin contacting DmOrc2-HTH is displayed in **k**. The key residues involved in interactions are labeled.

In State I, in addition to its interactions with the AAA+ domains of ORC4 and ORC5 as seen in DmORC and ScORC structures^18–20^, ORC1-WHD also engages with α3 of the HTH motif from ORC2-WHD through its β-hairpin motif (Fig. 2a–c, g). These interactions also induce noticeable conformational changes in other ORC subunits (Fig. 2i, j). For example, compared with that in ORC2–5, ORC2-WHD in ORC1–5 State I undergoes a small rotation (Fig. 2j and Supplementary Movie S1). Additional domain movements can also be observed on ORC2-AAA+ and ORC3-WHD, rendering a slight enlargement of the ORC ring (Fig. 2i, j and Supplementary Movie S1). Together, these changes weaken the interactions between ORC2-WHD and the DNA-binding channel, as shown by a preliminary semi-quantitative analysis based on calculated buried surface, which is reduced from 1000 Å in ORC2–5 to 800 Å in State I of ORC1–5 (Fig. 3a, b, e, f). The weakened interface likely permits ORC2-WHD to sample more conformations. In State II, ORC2-WHD retreats from the inner surface of the ORC ring, generating a hollow channel (Fig. 2d–f, h, k, l and Supplementary Movie S2). Thereupon, a new interface is formed between the HTH motif of ORC2-WHD and the β-hairpin motif of ORC1-WHD (Figs. 2h and 3c, g). The limited resolution at this region did not allow an independent assignment of the side chains contributing to this interface. However, the involved motifs are highly conserved among the metazoan ORCs (Fig. 3i, j and Supplementary Fig. S6o, p), a comparative analysis of the interface could be deduced from the crystal structure of DmORC (Fig. 3k, l). In DmORC, the equivalent residue of HsORC2-Phe524, that is Orc2-Phe566, is inserted into another hydrophobic cavity formed by five residues (Leu905, Ile892, Glu894, Lys903 and Glu851) from the β-hairpin of DmOrc1-WHD (Fig. 3i–l). The neighboring Arg563 and Glu564 of DmOrc2 interact with Asn907 and Arg889 of DmOrc1, respectively (Fig. 3k, l). Most likely, similar interactions in HsORC1–5 will be employed to stabilize ORC2-WHD in the conformation of State II. It should also be noted that the buried surface of ORC2-WHD in the central channel is reduced from 1000 Å in HsORC2–5 to 800 Å in State I of HsORC1–5 and then to 750 Å in State II of HsORC1–5 (Fig. 3e–g). The buried surfaces in State I and State II are comparable, suggesting that they are two inter-changeable equilibrium states (Fig. 3f, g).

Therefore, these observations suggest that the binding of ORC1 functions to transform HsORC2–5 from a tightly inhibited state into a partially activated conformation in a stepwise manner. The interaction between ORC1-WHD and ORC2-WHD induces the transition from State I to State II, which is likely the first step of ORC activation for origin DNA binding.

### ORC1-AAA+ domain in ORC1–5 is highly dynamic

**Fig. 4:**
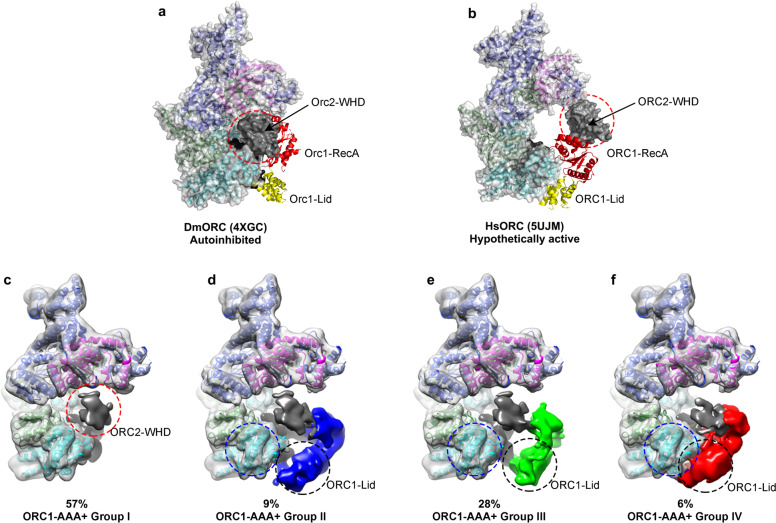
Conformational dynamics of ORC1-AAA+ domain in human ORC1–5. **a**, **b** Crystal structure of the autoinhibited DmORC (PDB: 4XGC) (**a**) and a hypothetic atomic model of the active HsORC (PDB: 5UJM) (**b**) shown in cartoon and surface representations in AAA+ view. Orc2-WHDs are highlighted by red dash circles. **c–f** Cryo-EM maps of four subgroups, groups I (**c**), group II (**d**), group III (**e**), group IV (**f**), classified based on the conformations of ORC1-AAA+. ORC1-AAA+ domains are colored blue (**d**), green (**e**), and red (**f**), respectively. ORC2-WHDs are colored dark gray. Densities of other domains are shown in transparent surface representation and superimposed with the atomic model of HsORC1–5. The positions of ORC4-RecAs and ORC1-Lids are marked by blue and black dash circles, respectively.

ATP binding by ORC1 plays an important role in recruiting ORC onto origin DNA^10,51^, and the correct assembly of the ATPase center of ORC1:ORC4 is an important feature of final activation as observed in the structures of ScORC-DNA, ScOCCM, and HsORC1/4/5 motor module^19–21^. In the HsORC1–5 maps, ORC1-AAA+ appears highly dynamic and its density is highly fragmented and only visible at very low contour level (Supplementary Figs. S6e, f and S7b–d). It suggests that the ATPase center of ORC1:ORC4 is completely disrupted in HsORC1–5. Interestingly, although the Orc1:Orc4 ATPase center is also not correctly formed in DmORC structure (Fig. 4a), Orc1-AAA+ is stably attached to the WHD layer, albeit in a position very different from the active conformation. This discrepancy between HsORC and DmORC might be caused by experimental settings as X-ray crystallography can only resolve one conformation each time while cryo-EM is able to capture multiple states of a dynamic complex in a single sample. To address this issue, we focused on the region of ORC1-AAA+ to perform an unsupervised local 3D classification with HsORC1–5 particles (Supplementary Fig. S3b). We can identify three subgroups of particles (group II, III, IV) showing relatively stable ORC1-AAA+ especially for its lid subdomain (Fig. 4d–f and Supplementary Figs. S3b and S7f–h). The locations of ORC1-AAA+ in group II (9% particles) and III (28% particles) are similar as that in DmORC (Fig. 4a, d, e and Supplementary Fig. S7f, g). These results indicate that the autoinhibited form of ORC as observed in Drosophila represents only one conformational state which also exists in human ORC^18,48^. Notably, the particles from group IV (6% of particles) render a structure with ORC1-Lid subdomain correctly engaged with ORC4-AAA+, resembling an active ATPase pocket (Fig. 4b, f and Supplementary Fig. S7h). As also shown in the 2D average images, after engaging with ORC4-AAA+, ORC1-Lid becomes more ordered with discernible secondary structural features (Supplementary Fig. S7h).

Together, these results indicate that after the association of ORC1 with ORC2–5, ORC1-AAA+ remains flexible and is free to assume different positions. The conformation of HsORC1–5 in group IV might represent an intermediate that is readily to be converted into the next stage as a fully activated ORC.

### Coordinated domain rearrangement to regulate the size of the central channel for DNA binding

It is evident that ORC activation involves at least two major conformational changes: (1) changes of ORC1-AAA+ to form an active ATPase center in the ORC1:ORC4 interface and (2) changes of ORC2-WHD to create a gate in the ORC ring for DNA entry. As revealed in HsORC1–5 State II, the central channel is still too narrow to accommodate the duplex DNA. Thus, to activate human ORC, additional domain movements are needed in order to enlarge the channel size for DNA to snug in (Supplementary Movie S3)^19,21^.

To better understand this process, we superimposed the structure of HsORC1–5 State II with available ORC structures in either autoinhibited (DmORC) or active (ScORC-DNA and hypothetic HsORC1–5) states^18,19,21^, using ORC4-RecA as a reference for alignment (Supplementary Fig. S8). To simplify the presentation, each domain (RecA, Lid, and WHD) was denoted by a point of mass center to illustrate their movement within both AAA+ and WHD layers upon ORC activation (Supplementary Fig. S8). As shown in Supplementary Fig. S8, the domain positions and the channel size in State II of HsORC1–5 are roughly the same as the autoinhibited DmORC (Supplementary Fig. S8c, f), whereas the sizes of the central channels in the two active forms are obviously larger than that in HsORC1–5 (Supplementary Fig. S8a, b, d, e and Movie S3). The comparison of our structure with the active ScORC–DNA indicates that most domains in HsORC1–5 have to undergo certain degrees of conformational changes in order to engage with dsDNA using its central channel. Among these changes, the movements of Orc2-RecA, Orc3-Lid, and Orc3-WHD are more significant (Supplementary Fig. S8a, d). Another interesting observation is that all Lid subdomains keep a similar moving pattern in both direction and distance with their neighboring RecA domains, Orc4-Lid with Orc5-RecA, Orc5-Lid with Orc3-RecA, and Orc3-Lid with Orc2-RecA, rather than with their own RecA subdomains (Supplementary Fig. S8a, b). This indicates that the Lid subdomain and the neighboring RecA act as a rigid body during structural rearrangements for ORC activation (Supplementary Fig. S8a, b).

## Discussion

In this study, we determined the cryo-EM structures of HsORC in two functional forms, ORC2–5 and ORC1–5. Both complexes adopt autoinhibited conformations, and in particular, HsORC2–5 appears to be more compact with its DNA-binding channel completely occupied by ORC2-WHD when compared with HsORC1–5 and DmORC. The binding of ORC1 rearranges ORC2–5 core complex into a less inhibited state with a narrow central channel. These results provide critical insights for us to understand the mechanisms of ORC regulation in metazoan to restrict pre-RC assembly only within G1 phase. The level of HsORC2–5 core complex remains constant in the nucleus throughout the cell cycle, presumably serving as a reservoir for ORC recycling^38,39,45^. The tightly autoinhibited conformation of HsORC2–5 could be a stringent control to prevent the unlicensed origin activation. The recruitment of ORC2–5 onto origin DNA largely depends on ORC1^38,40,41,43^. However, upon ORC1 binding, ORC1–5 still remains in an autoinhibited state with ORC2-WHD sealing the central channel, suggesting that the loading of ORC on DNA might occurs in multiple steps, including at least two phases, an initial contact and a final encirclement. Indeed, besides the stable DNA encirclement via the central channel, ORC also has a transient DNA-binding mode^52,53^, and could even slide on DNA to search origin sites^54^. Therefore, our HsORC1–5 structures likely reflect the conformations upon initial contact of ORC with DNA, and the final encirclement may depend on DNA-induced conformational changes of ORC subunits.

**Fig. 5:**
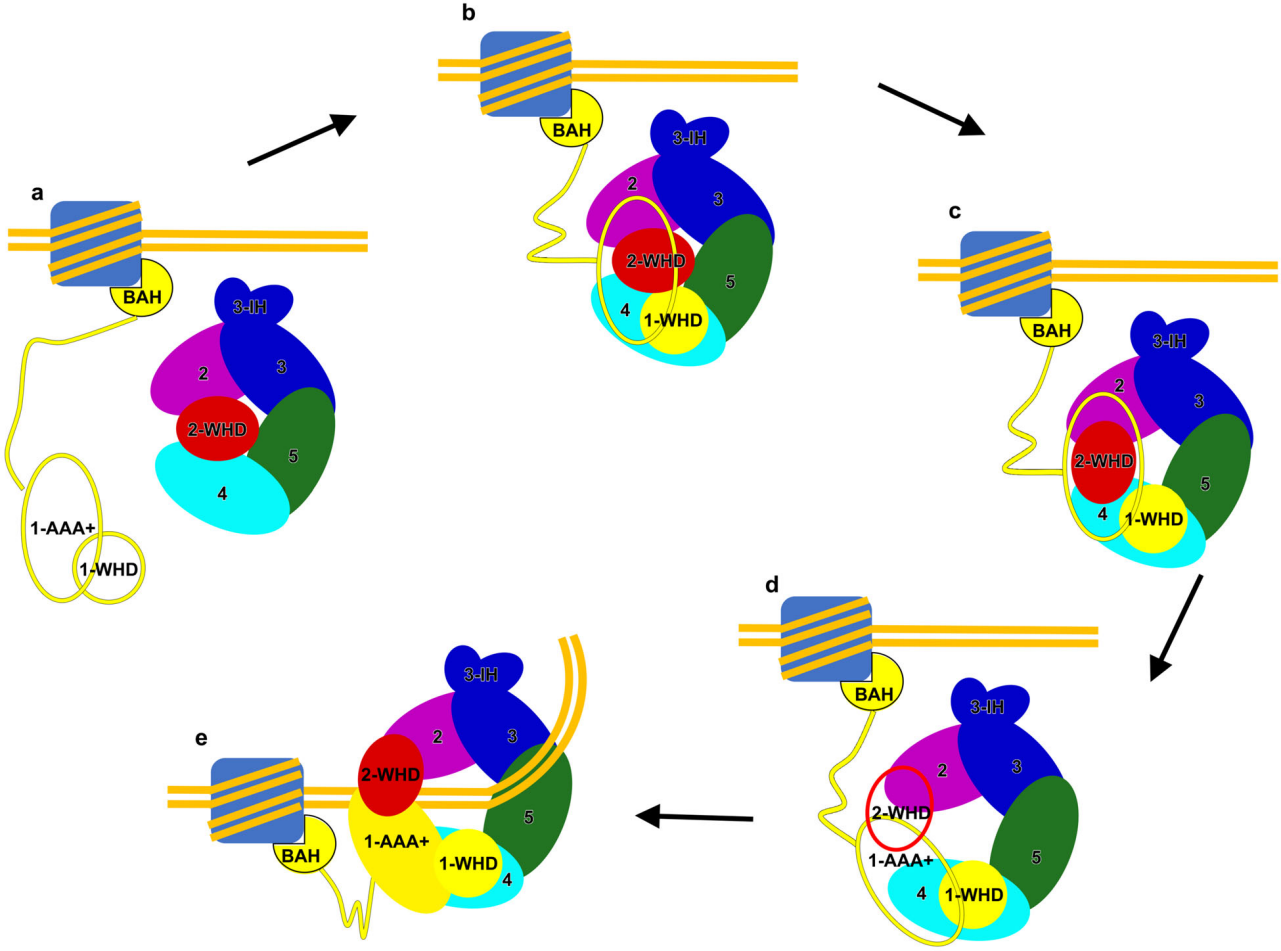
Updated model of step-wise assembly and activation of human ORC. **a** ORC1 recognizes and binds to the initiation site through its N-terminal BAH domain or other DNA-binding elements. **b** Chromatin-anchored ORC1 recruits ORC2–5 to form a tightly inhibited conformation (HsORC1–5 State I). **c** ORC1–5 transits to another conformational state (HsORC1–5 State II), still autoinhibited, but to a less extent. **d** Intrinsic flexibility of ORC1–5 (with flexible ORC2-WHD and ORC1-AAA+, denoted by unfilled red and yellow ovals, respectively) permits the sampling of fully active conformation that is competent for DNA encirclement. **e** ORC1–5 encircles DNA using the canonical DNA-binding channel and is converted to a functionally active form ready for CDC6 association and subsequent helicase loading.

Combining all published data, here we propose a model to illustrate possible mechanisms regulating HsORC activation and its subsequent recruitment at origin DNA (Fig. 5). First, the free HsORC1 clasps onto chromatin regions with designated epigenetic marks using its BAH domain (Fig. 5a)^24,27,55^. This step is supported by previous data that HsORC1 associates with chromatin independently even in telophase of M phase, ahead of ORC2 and other ORC subunits^38^. Next, ORC1 uses its WHD as a bait to catch one tightly inhibited ORC2–5 from the reservoir (Fig. 5b). As shown in the HsORC1–5 structure, ORC1-WHD strongly interacts with ORC2–5 (Fig. 2a–f and Supplementary Fig. S6b–d). In support of this hypothesis, it has been reported that ORC1-WHD (residues 783–861) is the only domain necessary for ORC1 to engage with ORC2–5 as an assembly^40,43^. Upon ORC1 binding, a series of coordinated structural rearrangements take place in AAA+ and WHD domains of ORC2–5 subunits (Fig. 5c, d), which together (1) convert ORC1–5 from a tightly autoinhibited state (HsORC1–5 State I) to a less compact but still inactive conformation (HsORC1–5 State II) through repositioning ORC2-WHD; (2) completely mobilize ORC2-WHD and ORC1-AAA+, as seen in the apo-ScORC structure^19^, to open the entry gate and expand the central channel. The last step is DNA encirclement (Fig. 5e); ORC assumes an active conformation ready for engagement with CDC6. Before the final settlement of ORC on origin DNA, besides the intensively studied ORC1-BAH domain, some other DNA-binding motifs of ORC subunits are also potential to facilitate the landing of ORC on DNA, such as the conserved basic patches from ORC1-NTE region and the FOXA-like motifs in ORC1-AAA+ domain^19,22,38,48^. ORC6, a transcription factor II B (TFIIB)-like factor (Supplementary Fig. S1a), can bind to DNA independent of ORC1^56,57^. It is possible that the joint efforts from ORC1 and ORC6 help to tightly anchor ORC1–6 to DNA (Fig. 5e). Moreover, a novel ORC-associated protein in human, ORCA, which associates with ORC2 throughout the cell cycle and stabilizes the ORC–chromatin binding, might also contribute to the landing of ORC on chromatin^58,59^. Similarly, Girdin, a recently identified replication initiation factor able to bind origin DNA and associate with ORC in human cells^60^, is also a candidate factor for the first step DNA binding. Further investigations are needed to elucidate the detailed roles of ORC1, ORC6, ORCA, Girdin and some unknown factors as well as chromatin in this entire ORC activation process.

## Materials and methods

### Plasmid construction

Full-length codon-optimized synthetic genes of human ORC1–6 subunits were cloned into MultiBac baculovirus expression system. To facilitate the optimization of expression and purification, multiple tags were added to different subunits: twin-strep SumoStar tag at the N-terminus of ORC1, 3× Flag-tag at the N-terminus of ORC2, HA-tag at the C-termini of ORC3 and ORC5.

### Expression and purification of human ORC

Bacmid was generated in DH10Bac cell. Baculovirus was amplified three times before infection. *Sf21* insect cells were infected with baculovirus and cultured in SIM SF media (Sino Biological Inc.) for 48 h. Briefly, cells were collected and washed with ice-cold 1 × PBS first, and resuspended in lysis buffer A (25 mM HEPES-KOH, pH 7.6, 0.2 M KCl, 5 mM ATP, 10% glycerol, 0.05% (v/v) NP-40, 1 × PI cocktail). Following sonication, the lysate was centrifuged for 15 min at 100,000 × *g*. The supernatant was incubated with M2-flag affinity beads and eluted with 0.5 mg/mL flag peptide to obtain HsORC2–5 samples.

For HsORC1–5, cell pellets were firstly treated with high-salt lysis buffer B (25 mM HEPES-KOH, pH 7.6, 0.8 M KCl, 5 mM ATP, 10% glycerol, 0.05% (v/v) NP-40, 1 × PI cocktail), then resuspended in lysis buffer C (25 mM HEPES-KOH, pH 7.6, 0.4 M KCl, 5 mM ATP, 10% glycerol, 0.05% (v/v) NP-40, 1 × PI cocktail). After sonification and centrifugation, the supernatant was incubated with 50% slurry of Anti-HA Affinity Beads (Smart-Lifesciences) for 2–3 h at 4 °C. The beads were washed extensively and eluted with 0.5 mg/mL HA peptide. HA eluate was then incubated with Streptactin Beads 4FF (Smart-Lifesciences) for 1 h. After washing thoroughly, the recombinant HsORC1–5 was eluted with buffer A (25 mM HEPES-KOH, pH 7.6, 0.2 M KCl, 5 mM ATP, 10% glycerol, 0.05% (v/v) NP-40, 1 × PI cocktail) containing 20 mM desthiobiotin.

### Electron microscopy

The eluted HsORC2–5 and HsORC1–5 samples were subjected to glycerol gradient centrifugation in presence of 0–0.025% EM-grade glutaraldehyde for GraFix^49^. The gradient was centrifuged in Beckman TLS55 rotor (Beckman Optima TLX ultracentrifuge) for 13 h at a speed of 83,000 × *g* at 4 °C. Peak fractions were collected and cross-linking reaction was quenched by 40 mM ice-cold Tris-HCl (pH 8.0). Fractions containing ORC were concentrated by ultra-filtration. Glycerol was removed by buffer exchange. Negative staining by 2% uranyl acetate was used to confirm the sample homogeneity. Negatively stained grids were examined using an FEI Tecnai T20 electron microscope operated at 120 kV.

For cryo-grids preparation, aliquots (4 μL) of samples were applied to glow-discharged C-flat Au grids (R1.2/1.3, 400 mesh) inside the chamber of an FEI Vitrobot IV (4 °C and 100% humidity). Grids were flash frozen in liquid ethane, and screened using an FEI Talos Arctica microscope operated at 200 kV. Grids were then transferred to an FEI Titan Krios (operated at 300 kV) for data collection. Images were collected using a GIF K2 camera (Gatan) with SerialEM^61^ in the super-resolution counting and movie mode, at a nominal magnification of 165,000×, which renders a final pixel size of 0.415 Å at object scale (super-resolution), and with defocus ranging from –1 to –3.5 μm. A total of 32 frames were collected for each micrograph stack. The dose rate was 10.2 e^–^ s^–^ Å^–2^ with a total exposure time of 6.4 s.

### Image processing

For HsORC2–5 sample, the collected micrographs were manually screened and a total of 4678 qualified movie stacks were selected for image processing (Supplementary Fig. S2a). Drift-correction and electron dose-weighting were applied to movie stacks using MotionCor2^62^. Summed images with or without dose weighting were generated. Images without dose weighting was used to evaluate the parameters of contrast transfer function (CTF) by CTFFIND4^63^. To maximize the potential of the dataset, prior to the image processing on the whole dataset, the best 956 micrographs with higher quality were manually selected and subjected to pre-processing to generate the correct 3D reference, as well as the good 2D class averages as templates for more accurate particle auto-picking (Supplementary Fig. S2b). Following is the detailed procedures (Supplementary Fig. S2b, d). Around 1000 particles were manually picked to generate initial 2D averages for subsequent particle auto-picking, which generated 646 K particles from the selected 956 micrographs. 2D classification of these particles revealed large structural heterogeneity (Supplementary Fig. S2c). Only two class average images (with stable features of ORC subunits) were included as 2D templates for the second round of particle auto-picking (Supplementary Fig. S2c). This stringent standard led to a much-reduced number (262 K) of auto-picked particles (Supplementary Fig. S2b). With these particles, an initial 3D model was generated using RELION3.0. Further 3D classification kept 64 K particles for 3D refinement, resulting in a density map at overall resolution of 6.1 Å. To generate more 2D templates for particle picking, these 64 K particles were subjected to one round of 2D classification, which produced a collection of well resolved class averages. Subsequently, 1110 K particles were auto-picked from the whole dataset (4678 micrographs) with these updated and improved 2D templates (Supplementary Fig. S2d). After 2D classification, 750 K particles were subjected to one round of 3D classification (Supplementary Fig. S2d). The resulting eight classes contained similar numbers of particles (from 9% to 15%), and only one of them exhibited full structural features of ORC subunits. This class (108 K particles) was selected for 3D refinement with a global mask applied, resulting in a map at an overall resolution of 4.2 Å (Supplementary Fig. S2d). Application of CTF Refinement (particle-level local defocus) and Bayesian polishing in RELION3.0^64^ could improve the map to 4.1-Å resolution (Supplementary Fig. S2d). Non-uniform refinement with cryoSPARC^65^ further improved the map to 3.8-Å resolution (Supplementary Fig. S2d). Next, mask-based 3D classification on the region of ORC2-WHD with RELION3.0 was used to improve the local density. 52 K particles were selected for further refinement using cryoSPARC, resulting in a 3.9-Å map with better resolved ORC2-WHD (Supplementary Fig. S2d). All the resolution estimation was based on gold standard Fourier shell correlation at the cutoff of 0.143 (Supplementary Fig. S2g). The maps were sharpened by auto-evaluated B-factors. The local resolution map was generated using ResMap^66^ and displayed using UCSF Chimera (Supplementary Fig. S2e)^67^.

For the HsORC1–5 dataset, 5486 micrographs were manually selected out of the collected 6224 micrographs and similarly processed (Supplementary Fig. S3). A total of 588 K particles were selected after initial processing. Based on two rounds of 3D classification, 122 K particles were refined to 4.4 Å after Bayesian polishing. Supervised 3D classification (two references) was applied to separate two states of ORC2-WHD. The resolution was estimated to be 5.0 and 4.8 Å, respectively. As for ORC1-AAA+ domain, four groups were obtained through local-mask-based 3D classification.

### Model building

Each subunit of HsORC2–5 or HsORC1–5 was manually docked into the density maps with Chimera, using the available coordinates (PDB code: 5UJM). Subsequent model adjustment and rebuilding were done with Coot^68^. Models were further refined against the cryo-EM density maps using Phenix.real_space_refinement^69^ with geometry restraints and secondary structures restraints imposed. The refined atomic models were cross-validated to prevent over-fitting as previously described^70^. The atom coordinates were first randomized with a mean deviation value of 0.2 Å using the PDB tools in Phenix. The displaced models were refined against the Half1 map (produced from RELION refinement job) using Phenix.real_space_refinement with the same refinement parameters used above. FSC curves between the refined models and Half1 map (FSC_work_, model versus Half1 map), Half2 map (FSC_free_, model versus Half2 map), the final density map (model versus merge) were produced and compared (Supplementary Figs. S2h and S3e). The agreements of FSC_work_ and FSC_free_ curves indicated that all the ORC2–5 and ORC1–5 atomic models were not over-fitted. The buried surfaces between ORC2-WHD and the DNA-binding channel were calculated based on the atomic models using UCSF ChimeraX^71^. The mass centers of each domains were determined by the python script of PyMOL (center_of_mass.py, https://pymolwiki.org/index.php/Center_of_mass). Chimera, ChimeraX and Pymol (http://pymol.org) were used for figure preparation.

## Supplementary information

Supplementary information

Supplementary Movie S1

Supplementary Movie S2

Supplementary Movie S3

## Data Availability

Cryo-EM maps of the HsORC2–5, HsORC2–5 with improved local density of ORC2-WHD, HsORC1–5 State I, HsORC1–5 State II have been deposited to the Electron Microscopy Data Bank (EMDB) under accession numbers EMD-30462, EMD-30463, EMD-30464, EMD-30467, respectively. The atomic models of these three complexes have been deposited in the Protein Data Bank (PDB) with accession codes 7CTE, 7CTF, 7CTG.

## Supplementary information

**Supplementary Information** accompanies the paper at (10.1038/s41421-020-00232-3).
