# Supplementary material for: Structural insight into the assembly and conformational activation of human origin recognition complex
Source: Cell Discov. 2020 Nov 24;6:88. doi: 10.1038/s41421-020-00232-3 (PMC7684300; doi:10.1038/s41421-020-00232-3)
Supplement: Supplementary file 1 — Supplementary information [file 41421_2020_232_MOESM1_ESM.pdf]

Supplementary Materials for

Structural insight into the assembly and conformational activation of human origin recognition complex

Jiaxuan Cheng[1,2,†], Ningning Li[2,†,*], Xiaohan Wang[2], Jiazhi Hu[2], Yuanliang Zhai[3], and Ning Gao[2,*]

[1] State Key Laboratory of Membrane Biology, School of Life Sciences, Tsinghua University, Beijing 100084, China.

[2] State Key Laboratory of Membrane Biology, Peking-Tsinghua Joint Center for Life Sciences, School of Life Sciences, Peking University, Beijing 100084, China.

[3] School of Biological Sciences, The University of Hong Kong, Hong Kong, China

† Jiaxuan Cheng and Ningning Li contributed equally to this work.

* Correspondence: ningningli@pku.edu.cn (Ningning Li), gaon@pku.edu.cn (Ning Gao)

This file includes:

Supplementary Figs. S1 to S8

Supplementary Movie S1 to S3

Supplementary Table S1

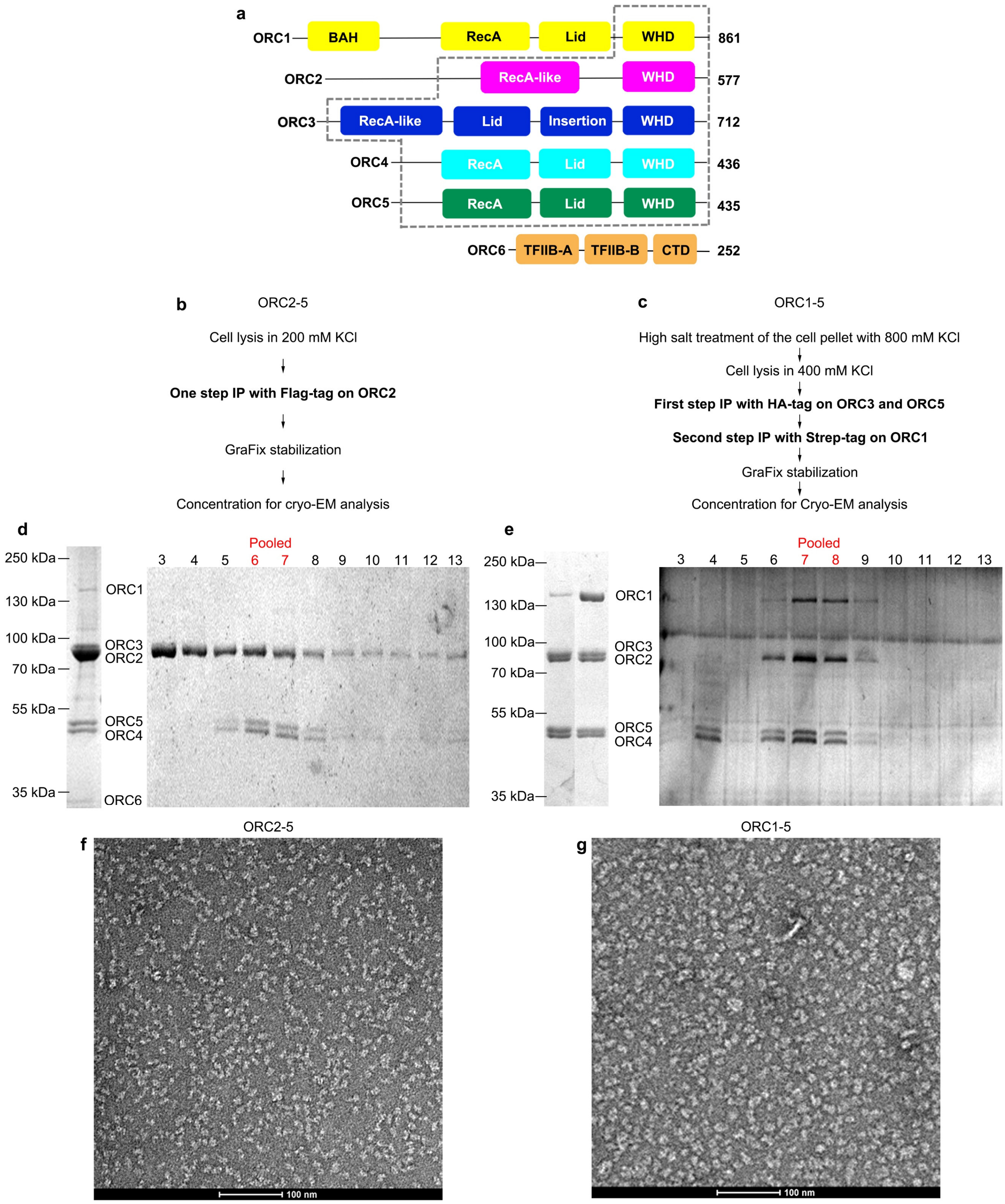

Supplementary Figure S1. Sample preparation of human ORC2-5 and ORC1-5 complexes.

(**a**) Schematic domain organization of ORC1-ORC6 subunits. Boxed region in dashed lines indicates the portions of ORC subunits that were well resolved in the final map and atomic models were built.

(**b-c**) Scheme for the preparation of ORC2-5 (b) and ORC1-5 (c) cryo-EM samples.

(**d**) SDS-PAGE analysis of the ORC2-5 sample. Left, affinity purification with Flag-tag on ORC2; Right, glycerol gradient centrifugation, fraction 6-7 were pooled for EM study.

(**e**) SDS-PAGE analysis of the ORC1-5 sample. Left, two-steps affinity purification with HA-tag on ORC3 and ORC5, and with twin strep-tag on ORC1; Right, glycerol gradient centrifugation, fraction 7-8 were pooled for EM study. Protein molecular weight makers, gel bands for ORC subunits and fraction numbers are labeled.

(**f-g**) Representative negative-staining EM images for GraFix-treated samples of ORC2-5 (f) and ORC1-5 (g).

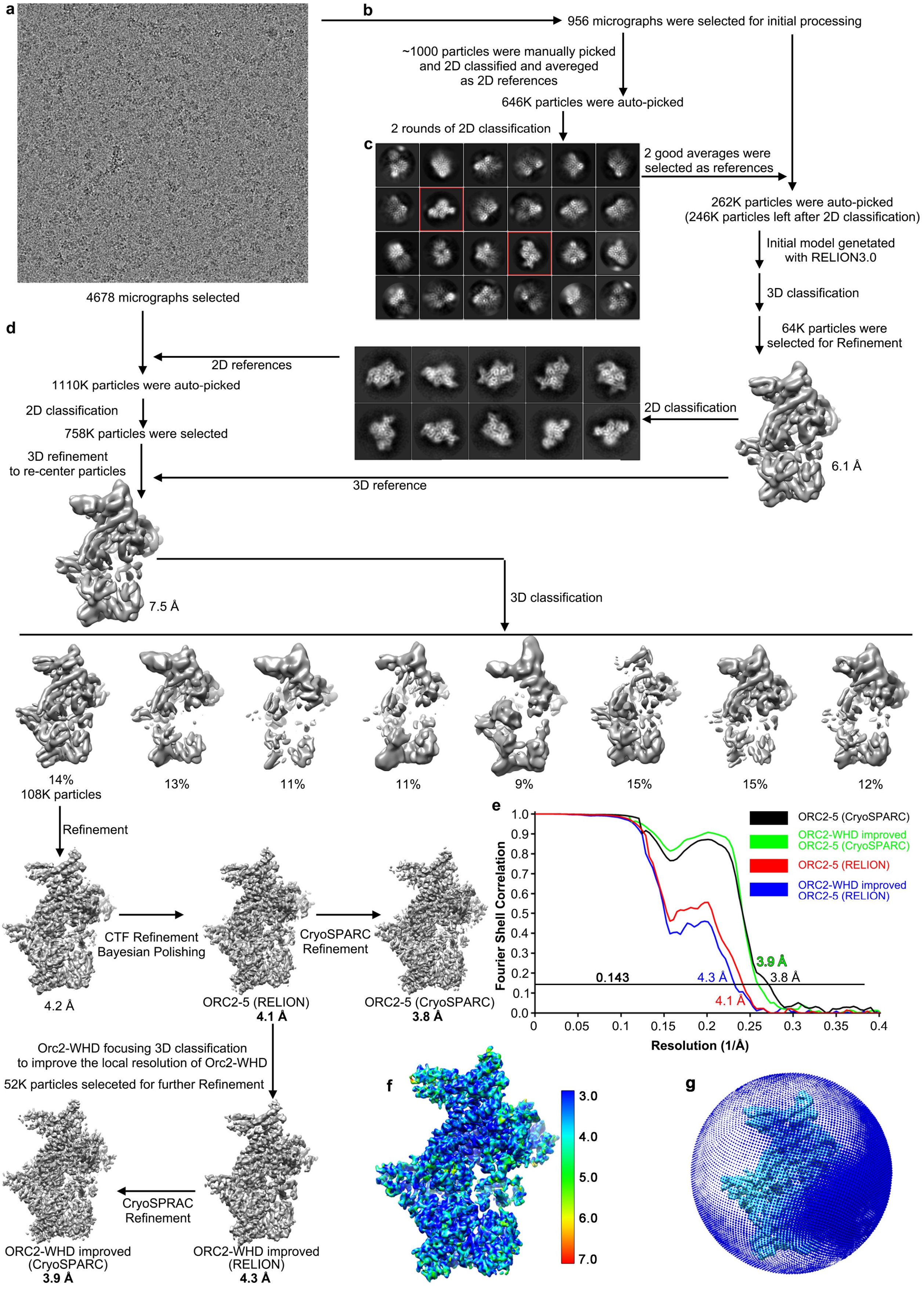

Supplementary Figure S2. Image processing of human ORC2-5 particles.

(**a**) A representative raw cryo-EM image of the ORC2-5 sample.

(**b-d**) Workflow of image processing of ORC2-5 particles (See Materials and Methods for details).

(**e**) Local resolution map of ORC2-5 at a global resolution of 3.8 Å.

(**f**) Angular distribution of particles from the final round of refinement for ORC2-5 (4.1 Å) using RELION3.0.

(**g**) FSC curves of density maps of ORC2-5 with final refinement using RELION3.0 and CryoSPARC.

(**h**) FSC curves for the atomic model cross-validation of ORC2-5.

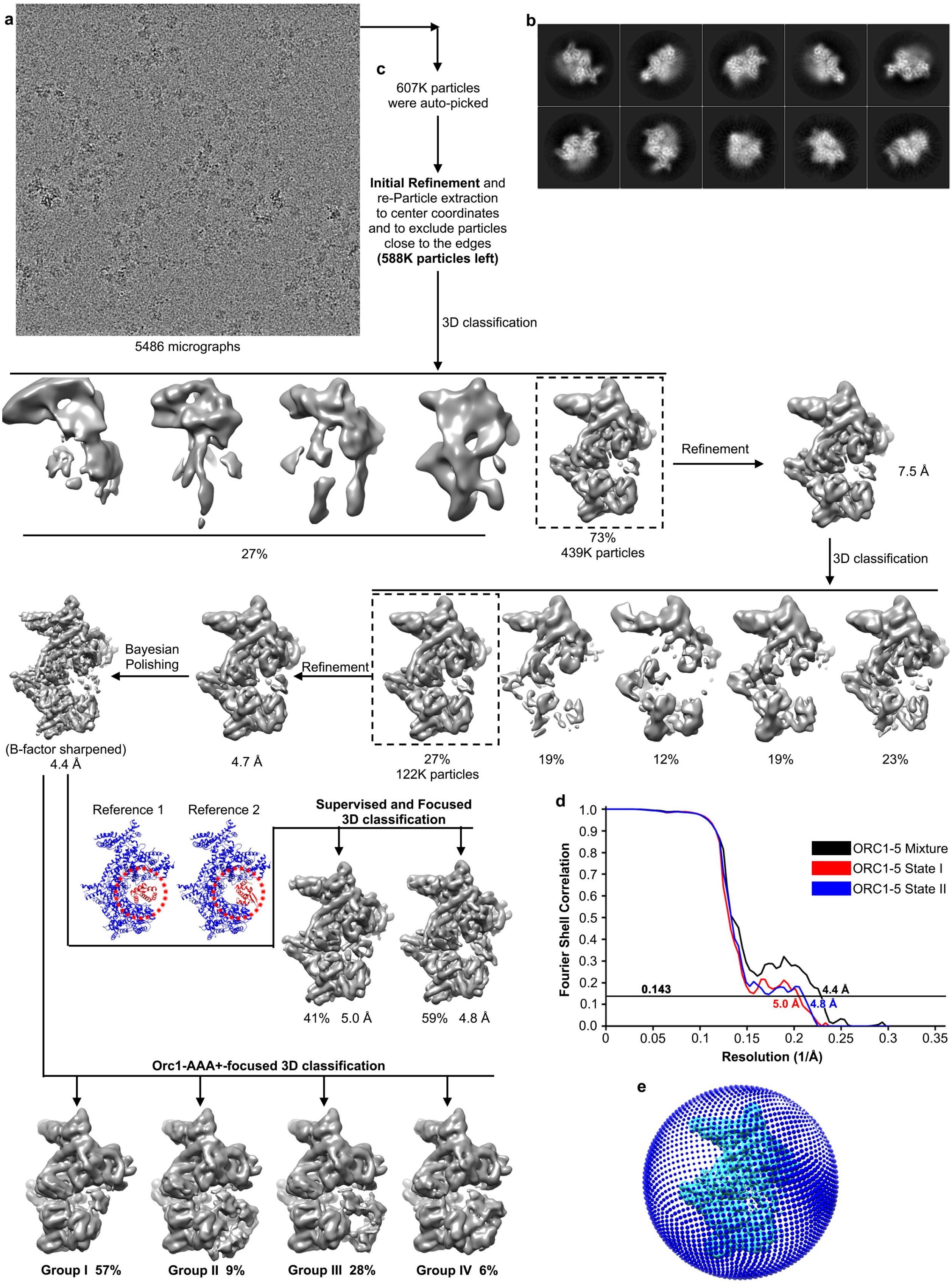

Supplementary Figure S3. Image processing of human ORC1-5 particles.

(**a**) A representative raw cryo-EM image of the ORC1-5 sample.

(**b**) Workflow of image processing of ORC1-5 particles (See Materials and Methods for details).

(**c**) Angular distribution of particles from the final round of refinement for the ORC1-5 mixture.

(**d**) FSC curves of the final maps of ORC1-5 mixture and the two separated conformational states, State I and II.

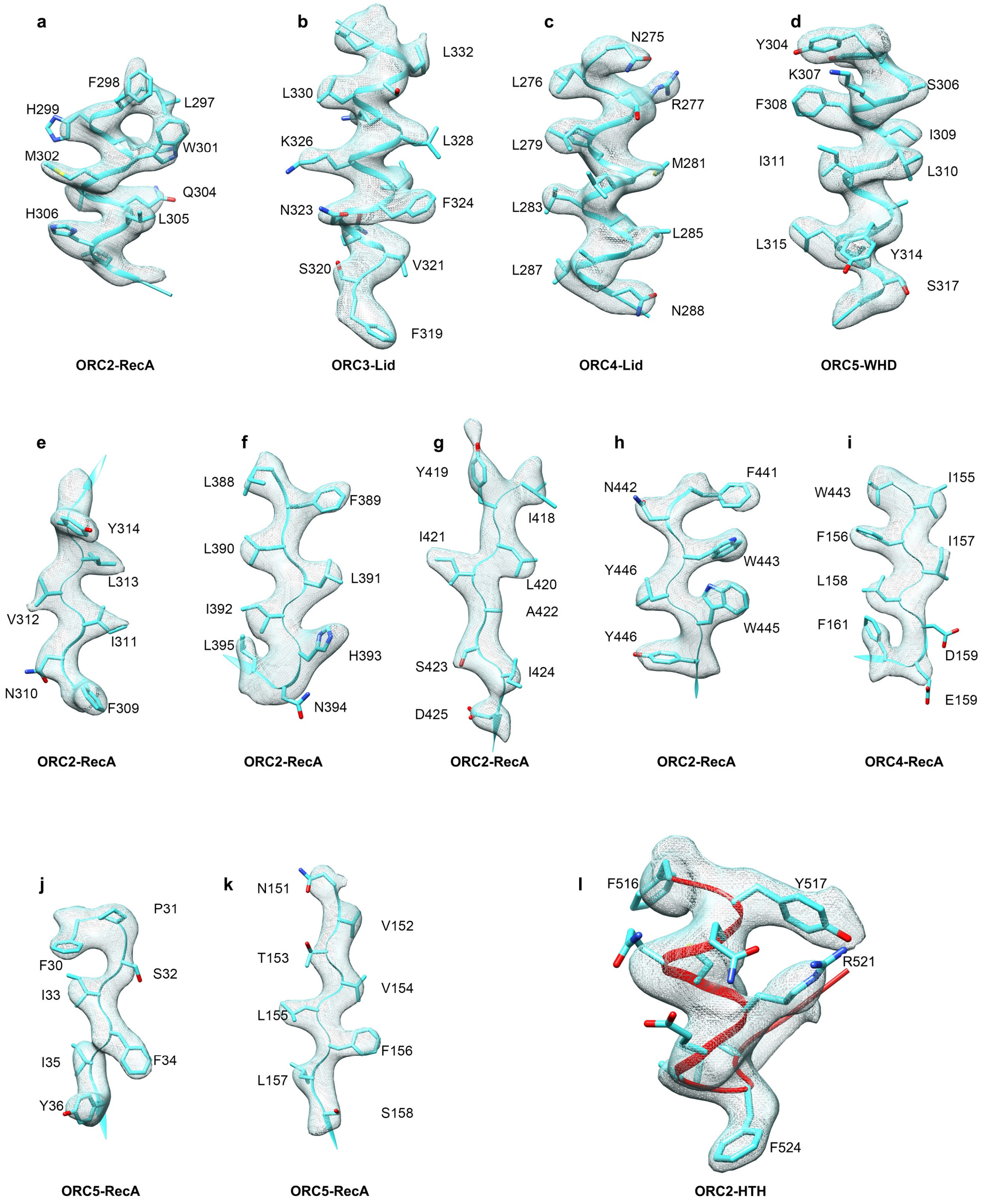

Supplementary Figure S4. Local density of representative regions of the cryo-EM map of human ORC2-5.

(**a-d**) Representative local densities of α-helices.

(**e-k**) Representative local densities of β-strands.

(**l**) Local density of the turn loop of ORC2-HTH.

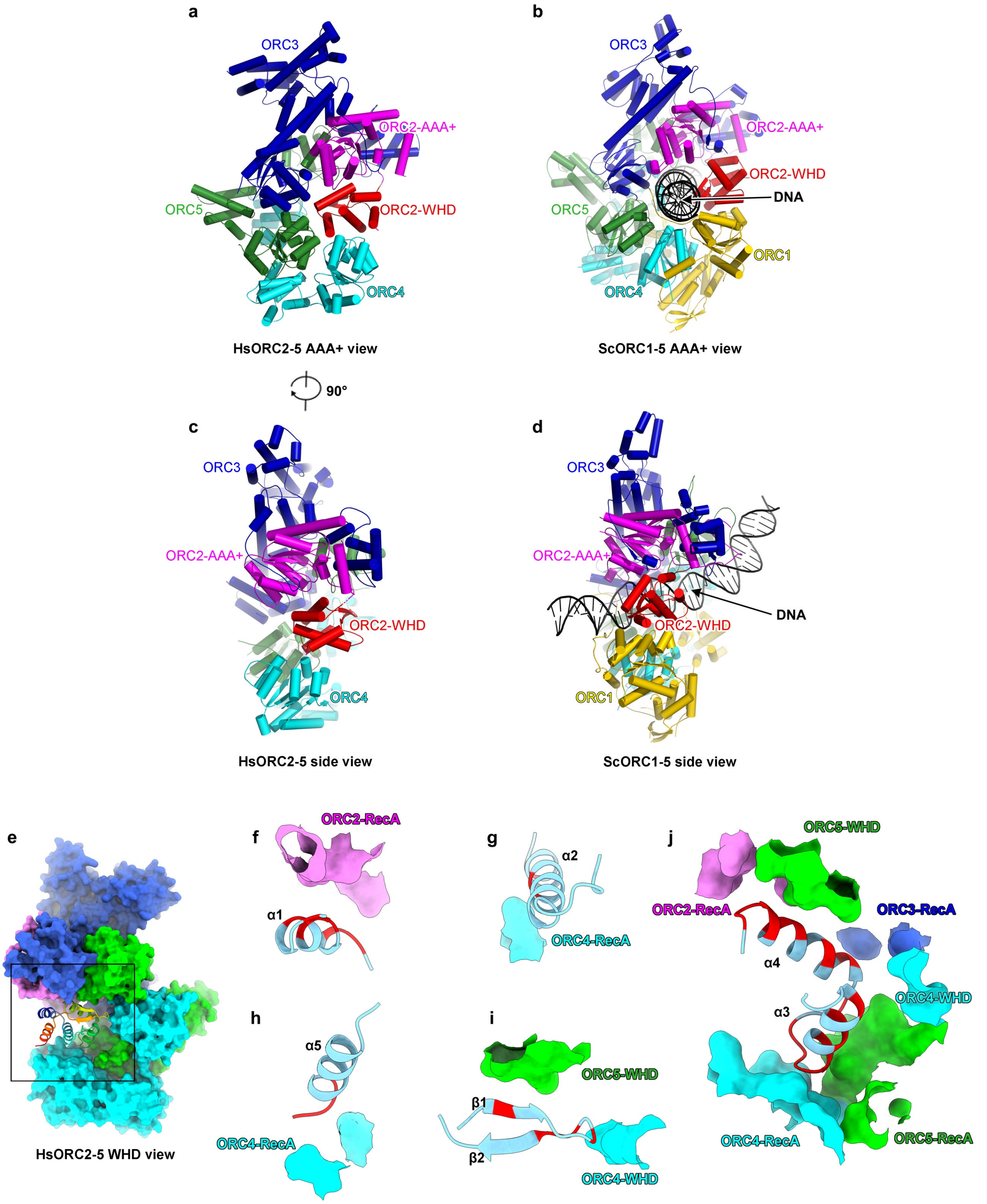

Supplementary Figure S5. Interaction of ORC2-WHD with the DNA binding central channel in human ORC2-5.

(**a-b**) Atomic model of HsORC2-5 (a) and ScORC bounded with DNA (b) displayed in AAA+ view.

(**c-d**) Same as (a-b), but displayed in side view.

(**e**) A thumbnail of atom model of HsORC2-5 in WHD view, with ORC2-WHD shown in cartoon and other domains in surface representation.

(**f-j**) Detailed interaction of the secondary structural elements of ORC2-WHD with the channel surface. The first α-helix (α1) of ORC2-WHD engages with ORC2-AAA+ (f), both α2 (g) and α5 (h) interact with ORC4-AAA+. Its β-hairpin motif (β1 and β2) makes contacts with the β-hairpins from ORC4-WHD and ORC5-WHD simultaneously (i). α3 and α4 stretch to the innermost surface of the channel and engages with all surrounding subunits (j). Each element of ORC2-WHD is shown in cartoon and the residues contributing to the buried surface are highlighted in red. The residues from the central channel components contributing to the buried surface are extracted and shown in surface representation.

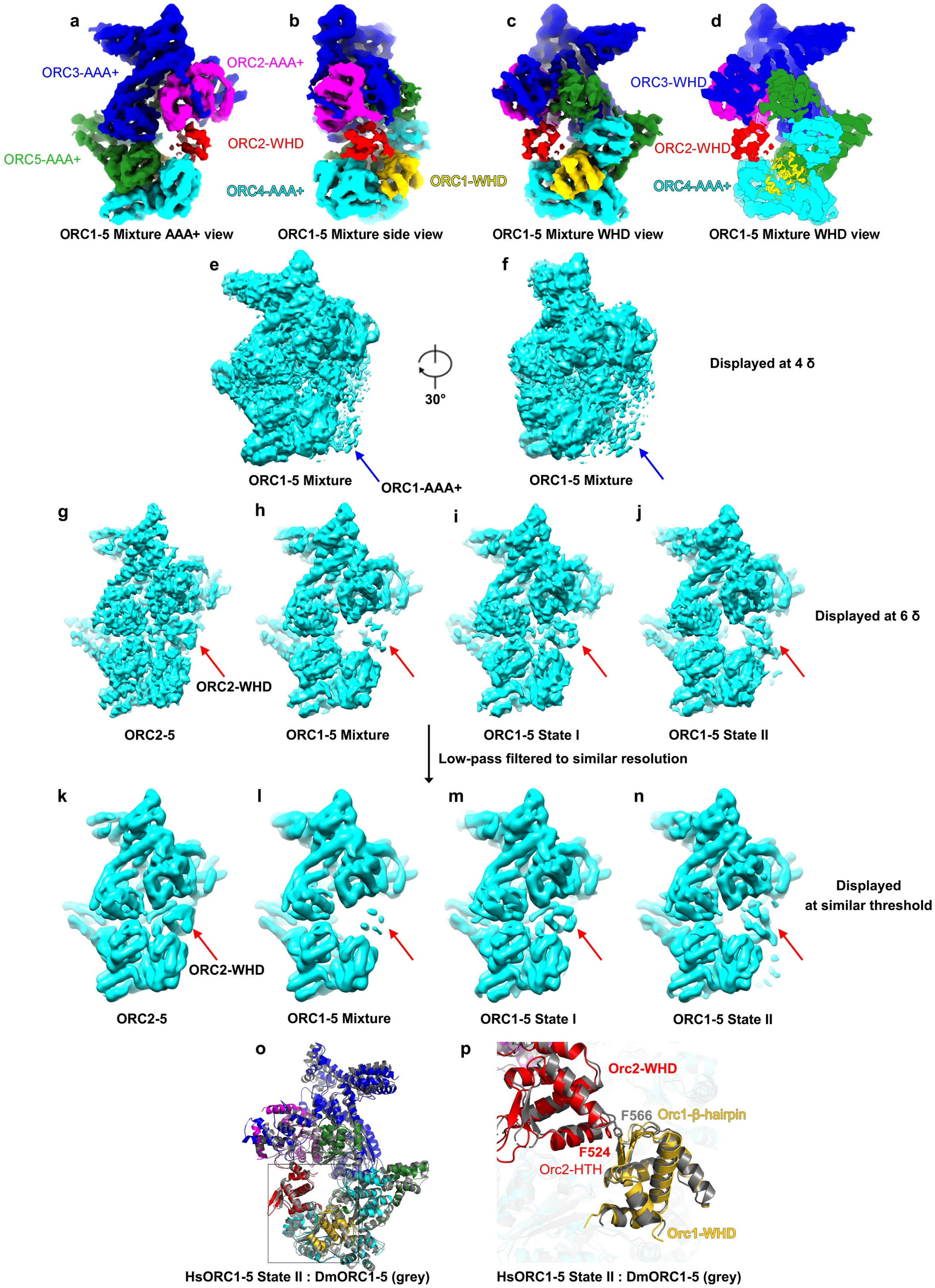

Supplementary Figure S6. Cryo-EM map of HsORC1-5 in the mixed state and the conformational dynamics of ORC1-AAA+ and ORC2-WHD.

(**a-d**) Cryo-EM density map of ORC1-5 mixture displayed in AAA+ view (a), side view (b) and WHD view (c-d). ORC subunits are color-coded and labeled. The local density of ORC1-WHD is replaced by its atomic model in (d) to show its interaction with AAA+ domains of ORC4 and ORC5.

(**e**) Cryo-EM density map of ORC1-5 mixture displayed at a contour level of 4σ in AAA+ view. The density debris of ORC1-AAA+ is indicated by a blue arrow.

(**f**) Same as (e), but with a 30° rotation.

(**g-j**) Cryo-EM maps of ORC2-5 (g), ORC1-5 mixture (h), ORC1-5 State I (i) and ORC1-5 State II (j) displaying at the same threshold (6σ) to show the occupancy of ORC2-WHD in the four structures. ORC2-WHD is highlighted by red arrows.

(**k-n**) Same as (g-j), but with maps low-pass filtered to similar resolution for better comparison.

(**o**) Superimposition of the atomic models of HsORC1-5 state II and DmORC. The atom model of DmORC is colored gray and HsORC is color-coded.

(**p**) Zoom-in view of the boxed region in (o), showing the interface of Orc1-WHD and Orc2-WHD. The HTH motif of ORC2-WHD and the β-hairpin motif of ORC1-WHD, and the pivotal residue of F524 are labeled.

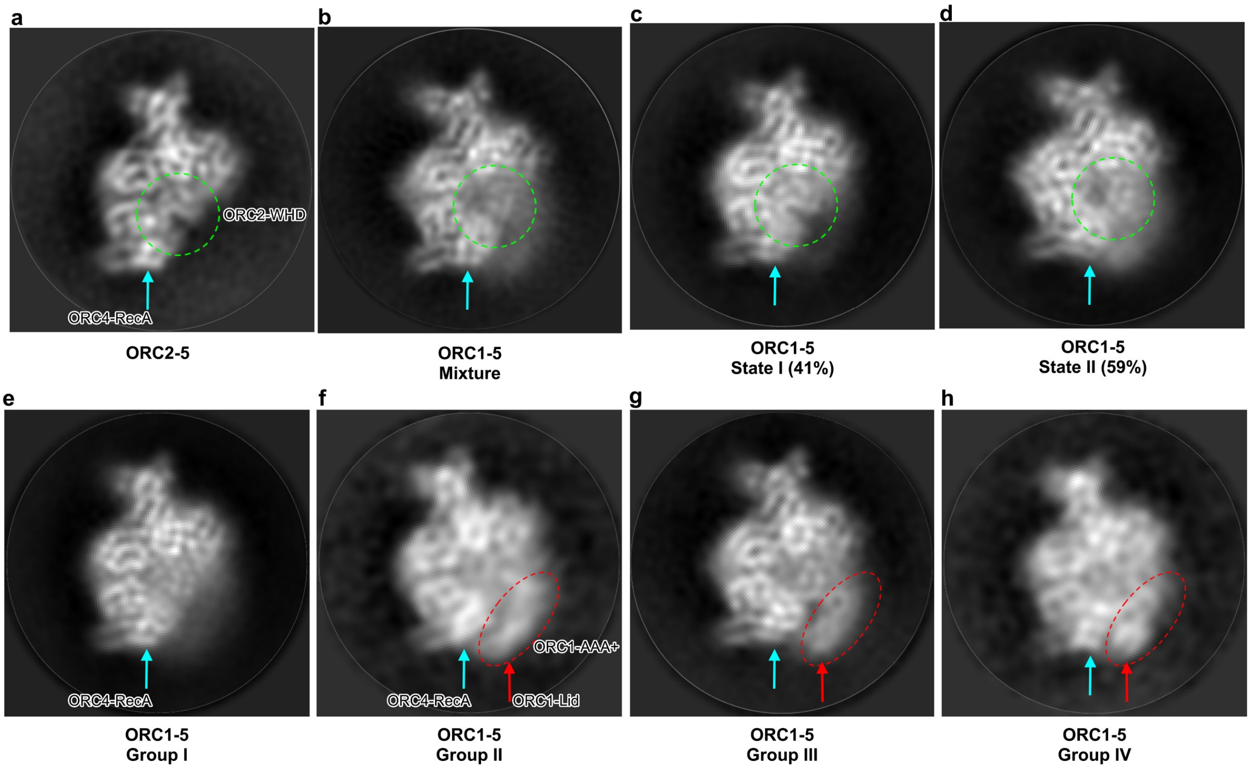

Supplementary Figure S7. Representative 2D class averages of different conformational states of human ORC particles.

(**a-h**) 2D class averages in AAA+ view of ORC2-5 (a), ORC1-5 mixture (b), ORC1-5 State I (c), ORC1-5 State II (d), and the four ORC1-5 subgroups classified based on ORC1-AAA+, group I (e), group II (f), group III (g), group IV (h). ORC4-RecAs are marked by cyan arrows in (a-h). ORC2-WHDs are highlighted by green dashed circles in (a-d). ORC1-AAA+ are highlighted by red dashed circles and ORC1-Lids are marked by red arrows in (f-h).

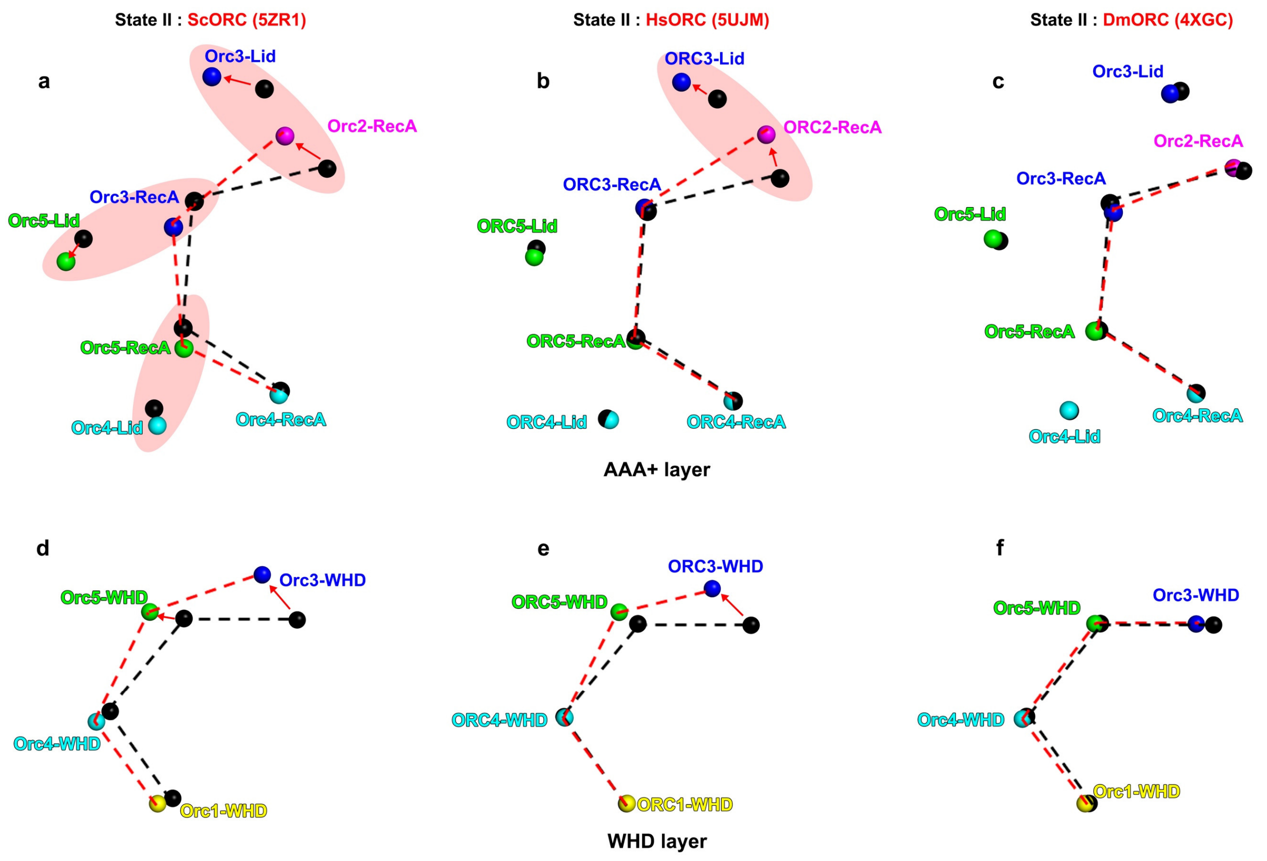

Supplementary Figure S8. Domain movement of ORC subunits upon transition from inactive to active conformations.

(**a-c**) Comparisons of the AAA+ layer of HsORC1-5 State II with ScORC (PDB: 5ZR1) (a), active HsORC (PDB: 5UJM) (b) and DmORC (PDB: 4XGC) (c). Each subdomain (RecA, Lid, WHD) is denoted as a point of mass center. Domains of HsORC1-5 State II are colored black and that of other structures are colored in the same color code as in (Fig. 2, a-f). The domains encircling the DNA duplex are linked using black (HsORC1-5 State II) or red (other structures) dash lines. The concerted movements of the Lid subdomain with the neighboring RecA are highlighted by red arrows.

(**d-f**) Same as (a-c), but showing comparisons of the WHD layers.

Supplementary Movies

Supplementary Movie S1: Domain movement of ORC subunits upon transition from ORC2-5 to ORC1-5 State I.

The structural transition from tightly autoinhibited ORC2-5 to autoinhibited ORC1-5 State I. Flashed ORC1-WHD shows the association of ORC1 to the core ORC2-5 complex. Atomic models are displayed in AAA+ view, WHD view and side view. The two atomic models are aligned based on ORC4-RecA. Note that a small but apparent dilation of the central channel could be seen during the transition.

Supplementary Movie S2: Domain movement of ORC subunits upon transition from State I to State II.

Same as Supplementary Movie 1, for the transition from ORC1-5 State I to State II. There is a rotation, ~120°, between the positions of ORC2-WHD in the two states.

Supplementary Movie S3: Domain movement of ORC subunits upon transition from autoinhibited to active conformations.

Same as Supplementary Movie 1, for the transition from autoinhibited ORC1-5 State II to hypothetic active conformation of HsORC (PDB:5UJM) (Rigid fitting of crystal structures of ORC1/4/5 and ORC2/3 into a 20-Å EM density map).

Supplementary Table S1

Cryo-EM data collection, refinement and validation statistics

	HsORC2-5 (EMDB-30462, 30467) (PDB 7CTE)	HsORC1-5 state I (EMDB-30464) (PDB 7CTG)	HsORC1-5 state II (EMDB-30463) (PDB 7CTF)
**Data collection and processing**			
Magnification	165,000X	165,000X	
Voltage (kV)	300	300	
Electron exposure (e–/Å^2)	65/dose weighting	65/dose weighting	
Defocus range (μm)	1-3.5	1-3.5	
Pixel size (Å)	0.83	0.83	0.83
Symmetry imposed	C1	C1	C1
Initial particle images (no.)	1110K	627K	
Final particle images (no.)	108K	49K	71K
Map resolution (Å)	3.8	5.0	4.8
FSC threshold	0.143	0.143	0.143
**Refinement**			
Initial model used (PDB code)	5UJM	5UJM	5UJM
Map sharpening *B* factor (Å^2)	-160	-156	-165
Model composition			
Non-hydrogen atoms	13699	14504	14504
Protein residues	1673	1775	1775
Ligands (ATP)	2	2	2
R.m.s. deviations			
Bond lengths (Å)	0.0126	0.0082	0.0089
Bond angles (°)	1.77	1.47	1.48
Validation			
MolProbity score	2.20	2.51	2.52
Clashscore	7.33	6.13	6.55
Poor rotamers (%)	2.93	8.90	8.72
Ramachandran plot			
Favored (%)	93.02	92.96	93.08
Allowed (%)	6.80	7.04	6.86
Disallowed (%)	0.18	0.00	0.06
